# Monocyte HLA-DR Assessment by a Novel Point-of-Care Device Is Feasible for Early Identification of ICU Patients With Complicated Courses—A *Proof-of-Principle* Study

**DOI:** 10.3389/fimmu.2019.00432

**Published:** 2019-03-12

**Authors:** Sandra Tamulyte, Jessica Kopplin, Thorsten Brenner, Markus Alexander Weigand, Florian Uhle

**Affiliations:** Department of Anesthesiology, Heidelberg University Hospital, Heidelberg, Germany

**Keywords:** SIRS, CARS, sepsis, infection, immunosuppression, tolerance, personalized medicine, precision medicine

## Abstract

**Background:** Critically ill patients, especially following trauma or extensive surgery, experience a systemic immune response, consisting of a pro-inflammatory as well as a counterbalancing anti-inflammatory response. Pro-inflammation is necessary for the initiation of homeostatic control and wound healing of the organism. However, when the counterbalancing mechanisms dominate, a condition of secondary immunodeficiency occurs, which renders the patient susceptible for opportunistic or secondary infections. However, the incidence of this condition is yet illusive.

**Methods:** For a period of 3 months (May to July 2017), 110 consecutive patients admitted to the surgical ICU of the Heidelberg University Hospital, a tertiary university hospital, were enrolled in the study. Monocyte HLA-DR (mHLA-DR), a long-known surrogate of monocyte function, was assessed quantitatively once on admission utilizing a novel point-of-care flow cytometer with single-use cartridges (Accelix system). Patients were followed up for further 28 days and data on ICU stay, antibiotic therapy, microbiological findings, and mechanical ventilation were recorded. Statistical analysis was performed to evaluate the incidence of immunosuppression—defined by different thresholds—as well as its consequence in terms of outcome and clinical course.

**Results:** Depending on the HLA-DR threshold applied for stratification (≤8,000/≤5,000/≤2,000 molecules/cell), a large group of patients (85.5/68.2/40.0%) already presented with a robust decrease of HLA-DR on admission, independent of the cause for critical illness. Analyzed for survival, neither threshold was able to stratify patients with a higher mortality. However, both thresholds of 2,000 and 5,000 were able to discriminate patients with longer ICU stay, ventilation time and duration of antibiotic therapy, as well as higher count of microbiological findings. Moreover, a mHLA-DR value ≤2,000 molecules/cell was associated with higher incidence of overall antibiotic therapy.

**Conclusion:** Single assessment of mHLA-DR using a novel point-of-care flow cytometer is able to stratify patients according to their risk of a complicated course. Therefore, this device overcomes the technical boundaries for measuring cellular biomarkers and paves the way for future studies involving personalized immunotherapy to patients with a high immunological risk profile independent of their background.

**Trial Registration:** German Clinical Trials Register; ID: DRKS00012348.

## Background

Extensive tissue injury, caused by either major surgery or trauma, induces a transient episode of sterile systemic inflammation, aiming to initiate damage and homeostatic control as well as wound healing (1–3). Simultaneously, a plethora of counterbalancing mechanism like, e.g., the apoptosis of lymphoid cells or the appearance of anti-inflammatory cytokines, occur. In their entirety, those are called compensatory anti-inflammatory response syndrome (CARS) (4). Its assumed evolutionary function is to prevent harm from overshooting inflammation, however, if this reaction is dominating it implies a higher susceptibility toward secondary and opportunistic infections with poor prognosis. In a nutshell, this acquired condition resembles a secondary immunodeficiency. Besides, the response pattern is skewed by the patient's predisposition, involving intrinsic factors as age, gender, and genetics as well as co-morbidities, the concomitant medications and lifestyle (5–7). Although antibiotic treatment can fight most of these arising infections, it cannot approach the fundamental problem of an impaired immunity of the host. Even worse, systemic antibiotic therapy can alter the composition of the body's microbiota, thereby opening niches for the colonization and expansion of opportunistic pathogens like, e.g., *Clostridium difficile* (8, 9). In sum, a vicious cycle between host and pathogens develops, implying tremendous harm to the organism.

An important objective is the development of host-directed therapies, aiming to restore the patient's endogenous immune capacity, especially at the boundaries of gut, skin, and airways (10). Despite a lack of approved drugs, several promising studies (predominantly with septic patients) have already been conducted evaluating the safety and feasibility of available immunomodulating compounds such as interferon-γ (IFN-γ) and granulocyte- or granulocyte/macrophage-colony stimulating factor (G-/GM-CSF) (11, 12). However, to fully exploit the benefit of these treatments and to avoid unnecessary exposure of critically ill patients to drugs and their side effects, patients need to be *a priori* stratified using robust and reliable surrogate biomarkers.

Various parameters have been assessed over the last 30 years, but the most prominent and widely used one for this purpose remains the downregulation of monocyte human leukocyte antigen-DR (mHLA-DR) (13, 14). As part of the heterodimeric major histocompatibility complex class II (MHC II) on the outer cell membrane, HLA-DR represents monocytes' capacity for antigen-presentation and by this means the crosstalk to T helper cells, enabling the activation of the adaptive immune system. Its predictive value concerning nosocomial infections and prognosis has been shown in clinical studies on various conditions, e.g., in patients suffering from sepsis (15), trauma (16, 17), burns (18, 19), or subjected to major surgical procedures such as liver transplantation (20, 21) or coronary artery bypass (22).

However, despite decades of research, mHLA-DR is rarely used in everyday clinical practice, due to the lack of broad access to flow cytometry and the availability of standardized assays. Also, as most previous studies focused on precisely defined groups of patients, there is little knowledge concerning the overall incidence of immunosuppression in the ICU. Consequently, the understanding of how many patients could benefit from personalized immunotherapy is limited. With newly emerging and miniaturized technologies in combination with simplified workflows, flow cytometry is finally coming to bedside and measurements can be facilitated by healthcare professionals around the clock at the point-of-care (POC) without the need of sample logistics and delayed results.

Making use of the Accelix system, a benchtop flow cytometer, our study aimed to determine the incidence of patients already presenting with decreased mHLA-DR already at ICU admission and the consequence of it regarding outcome and clinical course. We consecutively enrolled all patients admitted to a surgical ICU of a tertiary university hospital throughout 3 months. Quantitative mHLA-DR was measured once at admission, and the patients were followed up for further 28 days. As several thresholds have been reported before, we applied these to our cohort to delineate, which one projects best into complicated courses.

## Methods

### Study Design and Enrollment

Before enrollment of the first patient, the study protocol was assessed and positively evaluated by the local ethics committee (S-150/2017, Ethical Committee I of the Medical Faculty Heidelberg). Furthermore, the study was registered in the German Clinical Trials Register (ID: DRKS00012348). Over a period of 3 months (May to July 2017), all adult patients admitted to the surgical ICU of the Heidelberg University Hospital were enrolled in the study. Exclusion criteria were prior intensive care unit stays within the same hospital episode and the presence of therapy limitation/palliation at admission. Informed consent was obtained from the patient or, if not possible due to sedation or mental deterioration, from the legal representative. Cases without informed consent (*n* = 24) were excluded from the study's analysis.

On admission, all anamnestic data, as well as clinical scores and laboratory values (PCT, CRP, leucocytes), were obtained. Patients were followed up for a total of 28 days after admission (=day 0) and clinical variables (survival, antibiotic therapy, mechanical ventilation) were prospectively evaluated on a daily basis.

### Measurement of HLA-DR

Within 24 h from admission and within 2 h after blood draw, HLA-DR measurements were performed on a novel point-of-care flow cytometer (Accelix®, LeukoDX, Jerusalem, Israel). The system's characteristics and technical validity have been reported before (23). For each measurement, 40 μl of residual anti-coagulated blood drawn from the patient for routine blood gas analysis was applied onto the inlet port of a single-use cartridge containing antibodies as well as all reagents for cell preparation. After blood aspiration, the cartridge was closed and inserted into the Accelix® system for automated analysis within 25 min. The final HLA-DR value (average number of monoclonal antibodies bound per monocyte) was directly reported by the system after measurement. To facilitate this, an internal 4-point bead-based calibration curve was measured for each sample. Furthermore, as each anti-HLA-DR antibody is conjugated to only one molecule of fluorophore, the number of bound antibodies equals the number of molecules per cell.

In one case of a patient with severe neutropenia (0.3 leucocytes/nL), the system was not able to perform the measurement due to system-inherent algorithm-based quality rules, requiring a certain number of cellular events per second. The case was therefore excluded from the final analysis.

### Statistical Analysis

All statistical analysis and visualizations have been performed using SPSS Statistics (Version 25.0.0.1, IBM, Armonk, USA) with the exception of the scatter plot for individual HLA-DR values, which was generated in GraphPad Prism (Version 6.0c, GraphPad Software Inc., La Jolla, USA). Kaplan-Maier procedure was used for analysis of survival time and incidence of antibiotic therapy. Patients were grouped according to different thresholds of HLA-DR and groups were subsequently compared using the Log-rank test. Patients transferred or discharged from hospital were censored from the analysis of incidence of antibiotic therapy (detailed information of each patients' therapy was not legally permitted in case of treatment in other hospitals), but were maintained in the survival analysis, assuming no discharge in critical condition. For group comparisons of continuous variables (ICU stay, ventilation time, time under antibiotic therapy, microbiological findings), non-parametric Mann-Whitney *U* test was performed to compare different threshold groups. For the comparison of categorical variables, Chi-square test was performed. A *p-*value of ≤0.05 was accepted as significant for all comparisons. To assess the prognostic performance of HLA-DR *Area Under Receiver Operator Characteristic* (AUROC) analysis was performed regarding the variables “antibiotic therapy” and “28-day-mortality.” Area under curve (AUC) and the 95% confidence interval are reported as global indicators of discriminatory performance. To identify the cut-off value corresponding to the best combination of sensitivity and specificity, Youden index was calculated [(Sensitivity + Specificity)−1] and the maximum value selected.

## Results

### The Study Cohort and Incidence of Immunosuppression

**Figure 1:**
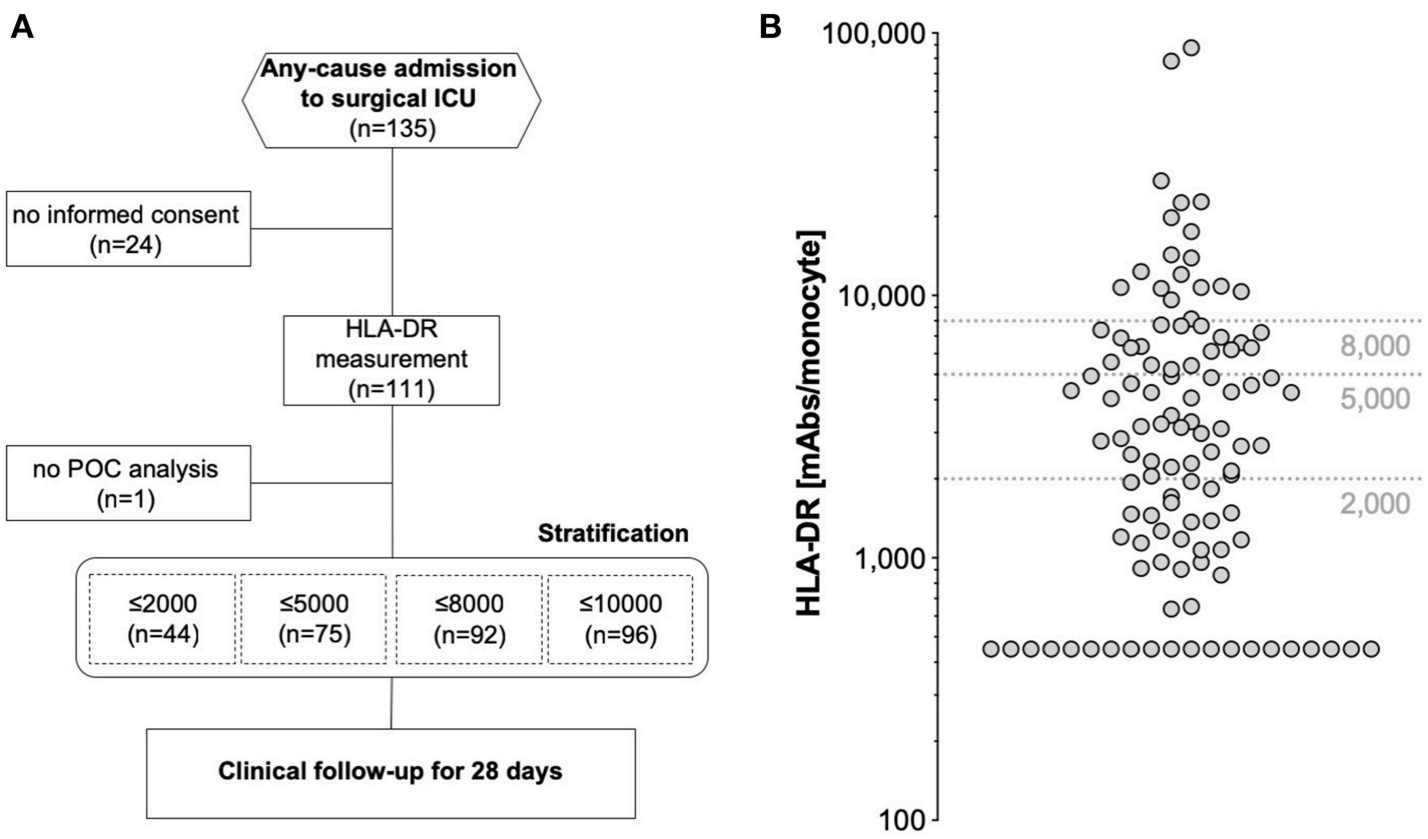
**(A)** Study flowchart according to STROBE and **(B)** distribution of HLA-DR measurements in the analyzed study cohort of 110 patients. Dashed horizontal lines depict threshold values indicated in earlier studies.

Overall, 135 critically ill patients consecutively admitted to a single surgical ICU of an academic hospital for any reason were enrolled. Of those, 110 were available for final analysis (24 patients dropped out due to inability to gain informed consent, one patient suffered from leucopenia and no POC HLA-DR assessment was possible) (Figure 1A). The median age of the study cohort was 63 years (range 20–92), with a majority of 83 male patients (75.5%) (Table 1). With nearly two thirds of patients grouped into ASA class III, the cohort exhibited a high burden of co-morbidities, especially of the cardiopulmonary system, and a high degree of illness on admission, as depicted by a median SOFA score of 5 (range: 0–17) and APACHE II score of 19 (range: 2–41) (Table 2). HLA-DR values only weakly, but yet significantly correlated with these scores (Supplementary Table 1). Only one patient after esophagectomy was admitted to the ICU in an elective manner, while the large majority of patients presented with either complications in the course of surgical treatment [unclear clinical deterioration: *n* = 42 (38.2%), surgery-associated infections: *n* = 19 (17.3%), bleeding complication: *n* = 9 (8.2%)], or came as external emergencies [*n* = 26 (23.6%)]. Multi-visceral resection [*n* = 28 (25.5%)], vascular as well as aortic surgery [*n* = 19 (17.3%); *n* = 13 (11.8%)] represented the most abundant procedures during the current hospital episode.

**Table 1:** Baseline demographic and medical information of study population.

	**ICU patients (*****n*** **= 110)**	
**DEMOGRAPHY**		
Age (years)	63	(20–92)
Sex (male)	83	75.5
**ASA CLASSIFICATION**		
I	6	5.5
II	22	20.0
III	69	62.7
IV	12	10.9
V	1	0.9
BMI (kg/m^2^)	25.45	(16.1–45.9)
**REASON FOR ICU ADMISSION**		
Preclinical emergency	26	23.6
Unclear clinical deterioration	42	38.2
Elective post-surgery	1	0.9
Infection (surgery-related)	19	17.3
Infection (other)	4	3.6
Bleeding	9	8.2
Internistic condition	4	3.6
Thromboembolic event	3	2.7
Other	1	0.9
**SURGICAL PROCEDURES**		
Esophageal resection	7	6.4
Gastrectomy	1	0.9
Small bowel resection	7	6.4
Colectomy	5	4.5
Liver resection	8	7.3
Pancreatic resection	1	0.9
Multivisceral resection	28	25.5
(Partial) Kidney resection	3	2.7
Bladder resection	1	0.9
Prostate resection	1	0.9
Liver transplantation	7	6.4
Aortic surgery	13	11.8
Vascular surgery	19	17.3
Polytrauma/Damage control surgery	20	18.2
Orthopedics	10	9.1
Other	40	36.4
**COMORBIDITIES**		
Diabetes mellitus	26	23.6
Coronary heart disease	20	18.2
Renal insufficiency	11	10.0
Liver cirrhosis	11	10.0
Inflammatory bowel disease	4	3,6
Leukemia	1	0.9
Hepatitis B	3	2.7
Hepatitis C	2	1.8
Peripheral arterial disease	7	6.4
Arterial hypertension	55	50.0
Atrial fibrillation	14	12.7
Dyslipoproteinemia	12	10.9
COPD	12	10.9
Asthma	2	1.8
Thyroid disease	15	13.6
Tumor	52	47.3
Other, cardiologic	10	9.1
Other	64	58.2

*All values represent number (%), except for age, and BMI, where median (min–max) is given. BMI of two cases could not be extracted from the medical records. ASA, American Society of Anesthesiologists; BMI, Body mass index; COPD, Chronic obstructive pulmonary disease*.

**Table 2:** Laboratory parameters, scores and outcome of study population.

	**ICU patients (*****n*** **= 110)**	
**LABORATORY PARAMETERS**		
Leucocytes (1/nL)	10.17	(1.58 – 39.4)
CRP (mg/L)	81.4	(1.9 – 428)
PCT (ng/mL)	1.37	(0.06 – 284.8)
**SCORES**		
APACHE II	19	(2 – 41)
SAPS II	33	(0 – 88)
SOFA	5	(0 – 17)
**OUTCOME**		
Antibiotic therapy on admission	55	50
Antibiotic therapy on day 1	60	54.6
Sepsis on admission	23	20.9
Length of ICU stay (d)	4	(1 – 29)
Ventilation time (d)	2	(0 – 29)
Cumulative antibiotic therapy (d)	9	(0 – 29)
Mortality (28-day)	11	10
Discharged (within observation time)	51	46.4

*All values represent median (min–max), except “Antibiotic therapy on admission/day 1,” “Sepsis on admission,” “Mortality,” and “Discharged,” where numbers (%) are given. CRP measurements are available of 109 cases and PCT of 60 cases. APACHE II, Acute Physiology And Chronic Health Evaluation II; CRP, C-reactive protein; PCT, procalcitonin; SAPS II, Simplified Acute Physiology Score II; SOFA, Sequential organ failure assessment score; ICU, Intensive care unit*.

On admission, 54 patients (49.1%) depended on mechanical ventilation, mainly due to lung failure [*n* = 41 (41.8%)] and half of the patients (*n* = 55) already received antibiotics when arriving in ICU. Sepsis (≥2 SIRS criteria + antibiotic therapy) was present in 23 patients (20.9%).

Quantitative monocyte HLA-DR expression was measured once after admission and obtained HLA-DR values ranged from below 450 HLA-DR molecules/monocyte (the system's lower limit of detection) to 87.768 molecules/monocyte (Figure 1B). We applied different thresholds reported earlier in literature to our results for further stratification: 94 patients presented (85.5%) with ≤10.000 molecules/cell [assumed as severe immunodepression (24)], 92 patients (83.6%) with ≤8.000 molecules/cell (25), 75 patients (68.2%) with ≤5.000 molecules/cell [postulated as threshold of immunoparalysis (24)], and finally 44 patients (40%) presented with ≤2.000 molecules/cell (26). In summary, our study involved unselected critically ill patients with a high degree of morbidity and with the majority of subjects already showing a lowered HLA-DR expression on monocytes on admission.

### Different HLA-DR Thresholds do Not Predict Survival

**Figure 2:**
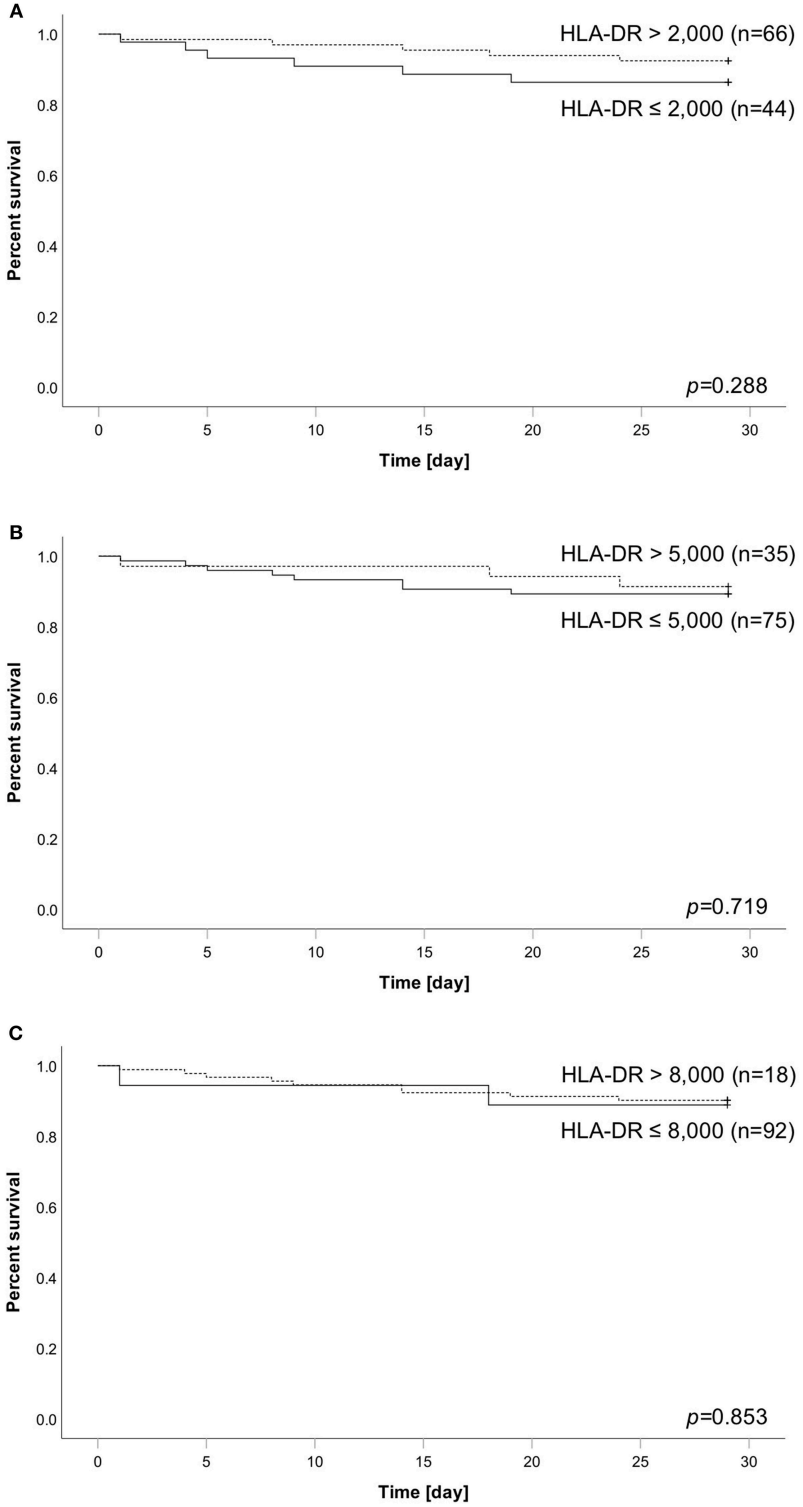
Analysis of patient survival over 28 days stratified according to HLA-DR thresholds of **(A)** 2,000, **(B)** 5,000, or **(C)** 8,000. Dashed line always indicates subgroup above corresponding threshold, whereas solid line indicates the group of patients below. Number in brackets equal subgroup size, a total of 110 patients has been analyzed. Group comparison was performed using logrank test and calculated *p*-values are given within the subpanels.

We applied different HLA-DR threshold (2.000/5.000/8.000) to our cohort and analyzed their association with survival. Overall, 11 patients (10%) died within the observational time frame. Concerning significance, none of the thresholds was able to stratify our cohort into groups of different survival outcome (Figures 2A–C). However, a threshold of 2,000 reached the best separation, with 13.6% (6 of 44) of patients below threshold dying within 28 days compared to only 7.6% of patients (5 of 66) with HLA-DR above threshold. A *de novo* AUROC analysis also found no HLA-DR cut-off with a predictive value for mortality in our cohort (Supplementary Figure 2).

### HLA-DR Is Able to Predict Complicated Clinical Courses

**Figure 3:**
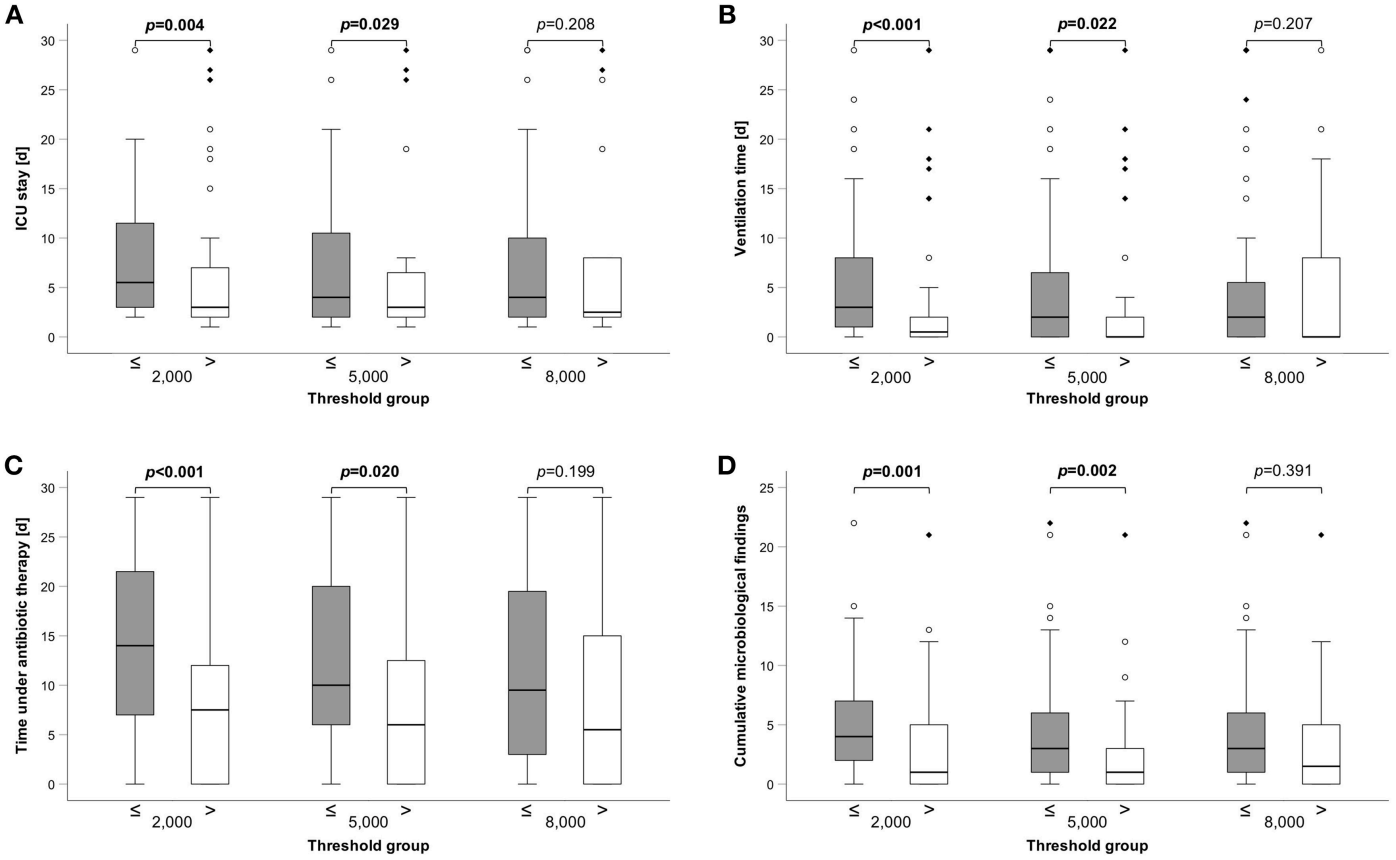
Group comparisons of clinical variables between different HLA-DR thresholds. **(A)** Length of ICU stay, **(B)** Ventilation time, **(C)** Time under antibiotic therapy (all 3 in days), and **(D)** Cumulative microbiological findings are shown. A total of 110 patients has been analyzed. Box edges represent quartiles with median given as horizontal line within, whiskers span the 95% confidence interval. Open circles and diamonds indicate outliers. Group comparisons were performed by Mann-Whitney *U*-test and *p*-values are given above the compared groups. Bold type indicates a *p* ≤0.05, assumed as significant.

Next, we applied the threshold and compared clinical variables between the groups. Patients with HLA-DR values ≤2,000 as well as ≤5,000 had a longer median ICU stay (6d (range: 2–29) vs. 3d (range: 1–29), *p* = 0.004; 4d (1–29) vs. 3d (1–29), *p* = 0.029) (Figure 3A, Supplementary Table 2). Similarly, duration of mechanical ventilation was longer in patients ≤2.000 and ≤5.000 HLA-DR: 3d (0–29) vs. 1d (0–29) (*p* < 0.001) and 2d (0–29) vs. 0d (0–29) (*p* = 0.022), respectively (Figure 3B, Supplementary Table 2). Severe infections are the superior threat on ICUs and we, therefore, analyzed the median antibiotic exposure time between HLA-DR stratified groups. Patients with HLA-DR ≤2,000 received systemic therapy for 14d (0–29) compared to 8d (0–29) (*p* < 0.001) and the patient group of HLA-DR ≤5,000 for 10d (0–29) compared to 6d (0–29) (*p* = 0.02) (Figure 3C, Supplementary Table 2). Not surprisingly, these results are corroborated by a higher number of total microbiological findings (for overview, see Supplementary Table 3) in the groups below thresholds [≤2,000: 4 (0–22) vs. 1 (0–21), *p* = 0.001; ≤5,000: 3 (0–22) vs. 1 (0–21), *p* = 0.002)] (Figure 3D, Supplementary Table 2). Surprisingly, when only blood cultures were considered, no difference was observed. In general, applying a threshold of 8,000 did not yield group of significant different outcomes. Importantly, disease severity as indicated by common ICU scores differed between the HLA-DR stratified groups, irrespective of the applied threshold (Supplementary Table 2). In conclusion, lower thresholds of 2,000 and 5,000 are capable to stratify patients into groups with longer ICU stay and ventilation time, longer antibiotic exposure and a higher number of microbiological findings.

### Lower HLA-DR Is Associated With Higher Incidence of Antibiotic Therapy

**Figure 4:**
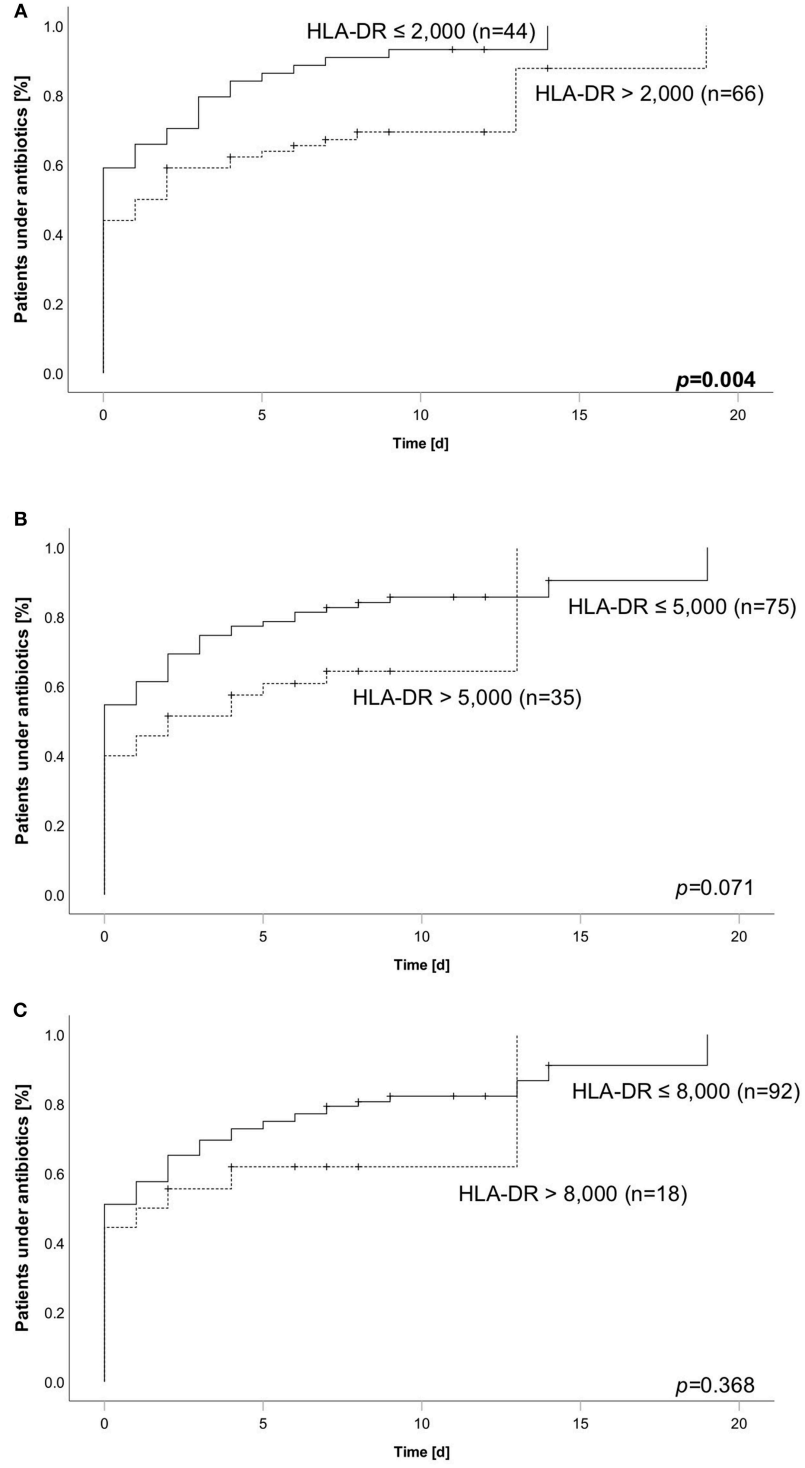
Cumulative incidence of antibiotic therapy over 28 days stratified according to HLA-DR thresholds of **(A)** 2,000, **(B)** 5,000, or **(C)** 8,000. Dashed line always indicates subgroup above corresponding threshold, whereas solid line indicates the group of patients below. Number in brackets equal subgroup size, a total of 110 patients has been analyzed. Group comparison was performed using logrank test and calculated *p*-values are given within the subpanels. Bold type indicates a *p* ≤0.05, assumed as significant.

Based on our finding of longer duration of antibiotics we asked about the fraction of patients needing antibiotic therapy within the observation period of 28 days. In line to the analysis reported before, we grouped the patients according to their HLA-DR value and different thresholds. We found an increased incidence when comparing patients below and above a threshold of 2,000 molecules/cell (95.5% (42 of 44 patients) vs. 74.2% (49 of 66 patients) (Figure 4A). For the thresholds of 5,000 and 8,000, no differences could be shown (Figures 4B,C). As half of the patients already received antibiotic therapy when admitted to ICU, we performed another analysis only including patients being antibiotic naïve on the admission day. Comparably, the lowest threshold of 2,000 significantly stratified patients according to their overall incidence of antibiotic therapy (88.9% (16 of 18 patients) vs. 54.1% (20 of 37 patients), while the others did not (Supplementary Figure 1). Aiming to evaluate the specific HLA-DR cut-off in our cohort, we conducted an AUROC analysis. We found a value of 4,266 to be of best predictive value [AUROC: 0.692 (0.573–0.811)] (Supplementary Figure 2), further substantiating the former results.

## Discussion

We report here the results of a proof-of-principle study on critically ill patients, using for the first time a novel point-of-care flow cytometer for easy and rapid assessment of mHLA-DR expression. We evaluated the incidence of decreased HLA-DR in an unselected cohort of patients immediately after ICU admission and found, independent of the reason for admission, a large percentage of patients presenting with dysfunctional monocytes, as defined by HLA-DR values below 2,000 or 5,000. Furthermore, those low values projected into the clinical course with “low HLA-DR” patients exhibiting a longer ICU stay and prolonged mechanical ventilation as well as antibiotic therapy.

Three decades ago, mHLA-DR entered the stage for the outcome prediction of trauma patients. At that time, the percentage of HLA-DR^+^ monocytes was incorporated into a score as a weight factor (27). Since then, more than 130 clinical studies conducted on a variety of patient cohorts have described the ability of mHLA-DR for prediction of outcome or secondary infection. However, until today, this parameter has not found its way into clinical practice for several reasons. Due to the lack of a standardized assay, mHLA-DR was reported in early studies either as “mean fluorescence intensity” (MFI; a raw parameter of cytometry) or as “HLA-DR^+^ monocytes.” Both parameters are largely influenced by a plethora of variables, including the preanalytic sample handling, antibodies, and protocols used for cell staining, and not finally the flow cytometer and applied settings. Not surprisingly, this heterogeneity hampers the comparability between the studies and, overall, the generalizability of the results. Despite this, interventional trials using IFN-γ were initiated very soon to correct for the low HLA-DR phenotype. Polk and colleagues examined in their RCT the efficacy of IFN-γ treatment in patients after trauma (28). They were able to show an increase in mHLA-DR^+^ monocytes, but no decrease in the incidence of major infections or death. Similarly, in a case series of nine patients with sepsis and <30% HLA-DR^+^ monocytes, IFN-γ treatment rapidly expanded this cell population (29).

Ten years later, this approach was repeated in a second case series of comparable patients (sepsis and MFI < 150), proving the ability of GM-CSF to reconstitute mHLA-DR (30). In 2005, a quantitative assay consisting of two antibodies (anti-CD14 and anti-HLA-DR; the latter conjugated in a fluorophore to antibody ratio of 1:1) and a separate bead-based calibration curve was developed, which enables the reproducible and platform-independent quantification of mHLA-DR as molecules per cell (24). Importantly, a comparable assay has been incorporated into the Accelix system used in our study, enabling technical comparability to other studies and machines (23). Döcke et al., did not solely develop the assay, but also reported two essential aspects: First, standard values of 100 healthy volunteers were reported, ranging from 13,200 to 42,500 for females (95% CI; median 26,200), and 15,300–40,100 for males (95% CI; median: 25,300). Those ranges were confirmed in a small cohort of 32 healthy donors, yielding a range of 13,255–20,890 (min–max; median: 16,884) (15). Similar to our study, they report a profoundly reduced expression of mHLA-DR in all subgroups of critically ill patients within 3 days of admission. This broad time frame to the first measurement was a result of limited laboratory availability, an archetypical barrier of implementation. In contrast, we assessed HLA-DR within 24 h after admission with no recruitment gaps. Astonishingly, if we would apply the standard values given above, only 9 of our 110 patients would possess “normal” mHLA-DR levels. Importantly, these numbers also clearly indicate that just being below standard range might not necessarily implicate an elevated risk for the individual patient. In the study of Lukaszewicz et al., a lack of HLA-DR reconstitution over time, assessed by several measurements, was predictive for secondary infection, but no association to mortality was observable. These core findings closely match our observations, despite the substantial differences in the study design. In line, Trimmel et al., were also not able to extrapolate outcome information from mHLA-DR assessment (31).

The second important achievement of Döcke et al., is the transfer of the old thresholds for “HLA-DR^+^ monocytes” (e.g., <30%) into their newly developed quantitative assay, thereby establishing a threshold of 5,000 molecules/cell indicative for an immunoparalysis. The implicated clinical risk for infection has been proven in a cohort of patients undergoing cardiac surgery with cardiopulmonary bypass (22). Overall, the informational content of mHLA-DR largely depends on the examined cohort: in contrast to total ICU patients, Wu et al., can delineate surviving from non-surviving patients with sepsis by comparing changes of HLA-DR^+^ monocytes over time (32). The rationale for this might lie in the bold difference of mortality between patients with sepsis and general ICU patients and the implicated effect size. In our cohort, we observed a 28-day mortality of 10%, in line with other reports (15). Compared to studies reporting overall ICU mortality (33), this value seems low and might be a consequence of delayed death beyond our observation time. Despite not significantly different, one could propose that with a larger sample size in our study, the threshold of 2,000 would have revealed slight mortality differences between the groups. However, when using a cellular biomarker of immune function for stratification of general ICU patients, the key question remains whether mortality is the relevant endpoint to look for or if endpoints of closer causality like, e.g., secondary infection, might be of higher interest.

In 2009, a small hallmark RCT on GM-CSF therapy for patients with sepsis used mHLA-DR values below 8,000 molecules/cell (for 2 subsequent days) as inclusion criteria and found GM-CSF to be able to reconstitute immune function as well as to decrease duration of mechanical ventilation (25). Despite a considerable heterogeneity of patients in our cohort containing only 23 patients with sepsis, we can readily observe a shorter time of mechanical ventilation in patients with mHLA-DR above thresholds of 2,000 as well as 5,000. Interestingly, a threshold of 8,000 in our cohort was not applicable for stratification. A technical and systematic bias can explain this: a value of 5,000 measured by Accelix is comparable to 8,000 molecules/cell on a conventional cytometer (23). The comparator of conventional cytometry was performed using the assay of Döcke et al., commercially available from Becton Dickinson under the brand name QuantiBrite. Considering the technical bias, it might be time to promote this assay from its informal status to “the” gold standard. This will enable to harmonize readouts of emerging systems like the Accelix in the future. Above this issue to be solved, one question remains: Which is the threshold of mHLA-DR to consider for patient stratification?

This dilemma can be symbolically pictured by two consecutive studies, which examined the usefulness of regulatory T cells, CD88 expression on neutrophils and mHLA-DR alone or in combination to predict infections in critically ill patients (26, 34). Both studies incorporated ROC analysis to find the optimal cut-off for prediction and while one of the studies revealed an optimal mHLA-DR cut-off of 10,000 molecules/cell, the concomitant INFECT study found a value of 2,009 to work best. Again, technical changes have been proposed as the underlying reason for this discrepancy. Our intention was not to identify novel thresholds, but we primarily made use of the previously reported ones [2,000 from (26), 5,000 from (15), and 8,000 from (25)] and applied it on Accelix-based measurements. We can clearly show that thresholds of 2,000 and 5,000 can identify patients of complicated clinical courses, most likely caused by nosocomial infections. However, if applying *de novo* AUROC analysis to our cohort, a cut-off of 4,266 works best to predict future antibiotic use in our patients. This approach further underlines the feasibility of the present cut-offs. As a limitation, our study did not approach the item “infection” directly as other studies did before. The reason for this is the challenge to group highly complex patients into this binary basket. Instead, we used “antibiotic therapy” as a surrogate. We anticipated that antibiotics would only be delivered to patients with clinically relevant infections (and stopped when feasible), as judged by the treating intensivists in daily practice. Nevertheless, we think our approach might even be more conservative, as our analysis for the incidence of antibiotic therapy does only consider the first initiated treatment and might mask further episode initiated later on. We can also clearly observe that subgroups of patients below threshold possess a higher severity of illness (e.g., SOFA score). HLA-DR might be proposed to serve as a pathophysiological bridge between critical illness *per se* and predisposition for infection.

In line with others, we propose low HLA-DR to be a good predictor for infection and complicated courses, but we should not anticipate that (secondary) infection necessarily projects into mortality when considering a general ICU population with high heterogeneity. Our results prove that immunosuppression is apparently a common feature of ICU patients and not an exclusive condition of sepsis or trauma patients. This concept is substantiated by a large-scale cohort study, which assessed the incidence of secondary infections between patients admitted either for sepsis or other reasons and found no differences (35). However, patients with sepsis and secondary infections exhibit tremendously higher mortality compared to non-sepsis patients, indicating the urgent need for risk mitigation.

Results of recent studies also indicate a potential value of HLA-DR for diagnosing sepsis in difficult-to-diagnose cohorts of patients in the emergency department or presenting with SIRS (36, 37). Importantly, the study by Parlato and colleagues did measure HLA-DR expression by quantitative PCR of whole blood samples, not by flow cytometry. This alternative approach has been reported before to be highly comparable to flow cytometry and if used on a digital PCR platform, to exhibit superior diagnostic performance to conventional biomarkers {Winkler:2017kl}{Almansa:2019ke}. However, if this approach is easier for real-life adoption and how it can be operationalized in terms of thresholds, especially in the context of immunosuppression, needs to be further elucidated.

Furthermore, the results of a recently completed confirmatory French RCT for GM-CSF treatment of sepsis patients with low mHLA-DR will finally evaluate the value of HLA-DR as a theranostic marker for immunotherapy (ClinicalTrials.gov identifier NCT02361528). Importantly, the primary endpoint of this trial is the incidence of secondary infection and, therefore, in case of a positive result, might be transferable to non-sepsis patients. To this end, the measurement of a biomarker makes only sense if it delivers actionable results to the clinician. However, when it comes to immunotherapy, many open gaps in knowledge remain to be filled. Which drug works best in which patient cohorts? For how long does a patient need to be immunosuppressed to involve a risk? And must it be an expensive and risk-associated intervention like pharmacological immunomodulation or might organizational and extended hygiene measures (e.g., strict access barriers) might already provide a patients' benefit?

In conclusion, assessment of mHLA-DR using the Accelix system, a novel point-of-care flow cytometer, is easy and reveals a broad incidence of immunosuppression in ICU patients. Furthermore, it is able to stratify patients according to their risk of a complicated course, including infection. Bedside systems can take away the work burden from central laboratories and by enabling rapid measurements on ward to circumvent HLA-DR-specific preanalytic problems that arise e.g., with extended storage of the blood. This class of devices is setting the future stage for stratification of patients into risk and therapy groups, enabling healthcare professionals to close the theranostic circle of immunotherapy.

## Data Availability

Data is available from the corresponding author on reasonable request.

## Author Contributions

ST designed the study and study protocol, obtained ethics approval, performed patient recruitment and informed consent, data collection, data analysis and discussion, and wrote the manuscript. JK performed measurements and data collection, and wrote the manuscript. TB performed patient recruitment and informed consent, and results discussion. MW designed the study and study protocol, obtained ethics approval, recruitment and informed consent, results, and discussion. FU designed the study and study protocol, obtained ethics approval, established laboratory methodology, data collection, analyzed the data, and wrote the manuscript. All authors read the manuscript draft and agreed upon its submission.

### Conflict of Interest Statement

The authors declare that the research was conducted in the absence of any commercial or financial relationships that could be construed as a potential conflict of interest.

## Abbreviations

APACHE: Acute Physiology and Chronic Health Evaluation
CD: Cluster of Differentiation
G-/GM-CSF: Granulocyte-/Granulocyte/Monocyte- Colony Stimulating Factor
HLA-DR: Human leucocyte antigen DR
ICU: Intensive care unit
IFN-γ: Interferon-γ
MFI: Mean fluorescence intensity
POC: Point-of-care
RCT: Randomized controlled trial
SOFA: Sequential organ failure assessment score.
