# Supplementary material for: Monocyte HLA-DR Assessment by a Novel Point-of-Care Device Is Feasible for Early Identification of ICU Patients With Complicated Courses—A Proof-of-Principle Study
Source: Front Immunol. 2019 Mar 12;10:432. doi: 10.3389/fimmu.2019.00432 (PMC6423155; doi:10.3389/fimmu.2019.00432)
Supplement: Supplementary file 4 [file Image_1.pdf]

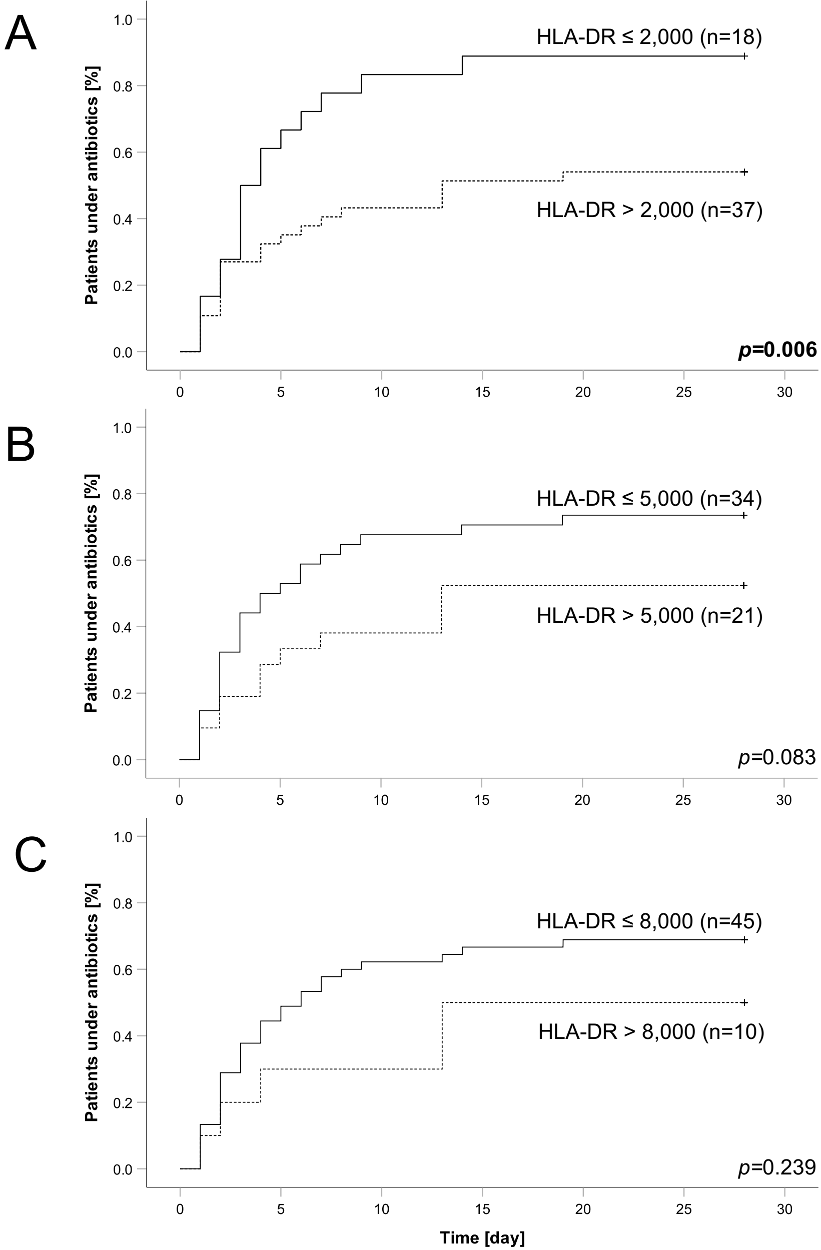

Supplementary Figure 1 Subgroup analysis of the cumulative incidence of antibiotic therapy over 28 days stratified according to HLA-DR thresholds of (A) 2,000, (B) 5,000 or (C) 8,000. Only patients without antibiotic therapy on admission were included in this analysis (n=55). Dashed line always indicates subgroup above corresponding threshold, whereas solid line indicates the group of patients below. Number in brackets equal subgroup size. Group comparison was performed using Log-rank test and calculated p-values are given within the subpanels. Bold type indicates a *p*-value ≤ 0.05, assumed as significant.
