# Supplementary material for: Monocyte HLA-DR Assessment by a Novel Point-of-Care Device Is Feasible for Early Identification of ICU Patients With Complicated Courses—A Proof-of-Principle Study
Source: Front Immunol. 2019 Mar 12;10:432. doi: 10.3389/fimmu.2019.00432 (PMC6423155; doi:10.3389/fimmu.2019.00432)
Supplement: Supplementary file 5 [file Image_2.pdf]

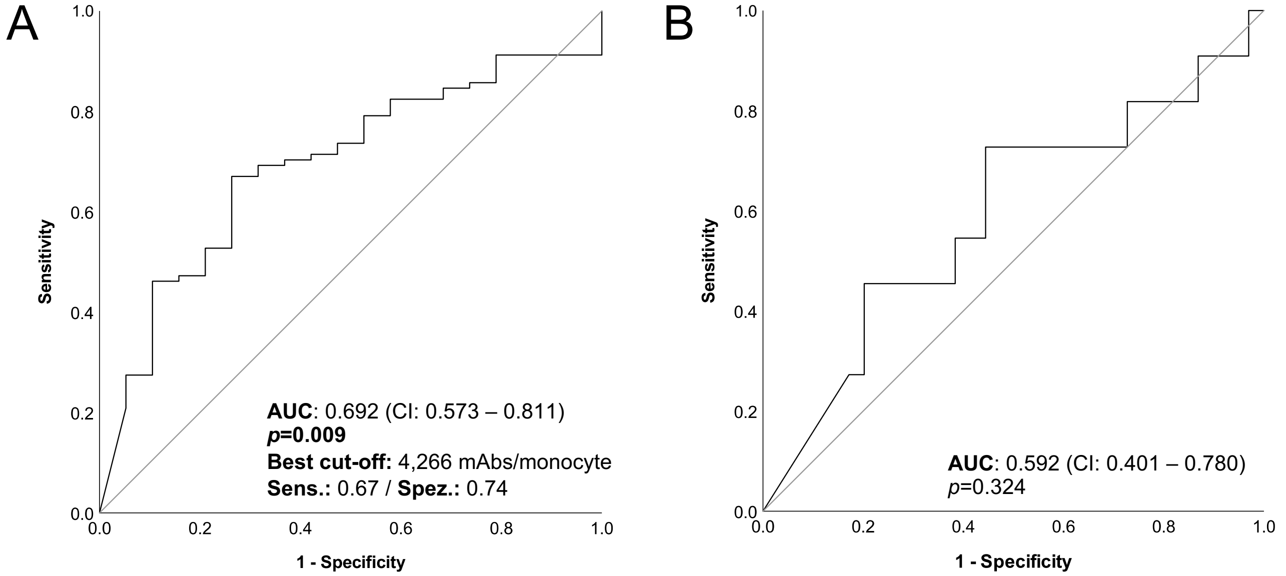

Supplementary Figure 2 AUROC analysis of HLA-DR to evaluate its prognostic value for (A) the initiation of antibiotic therapy, or (B) 28-day mortality. Best performing HLA-DR cut-off (and corresponding values for sensitivity and specificity) was determined for antibiotic therapy only by using the maximum Youden index ((Sensitivity+Specificity)-1). AUC: Area under curve. CI: 95% confidence interval. *p*-values are given within the subpanels, with $p<0.05$ judged as significant.
